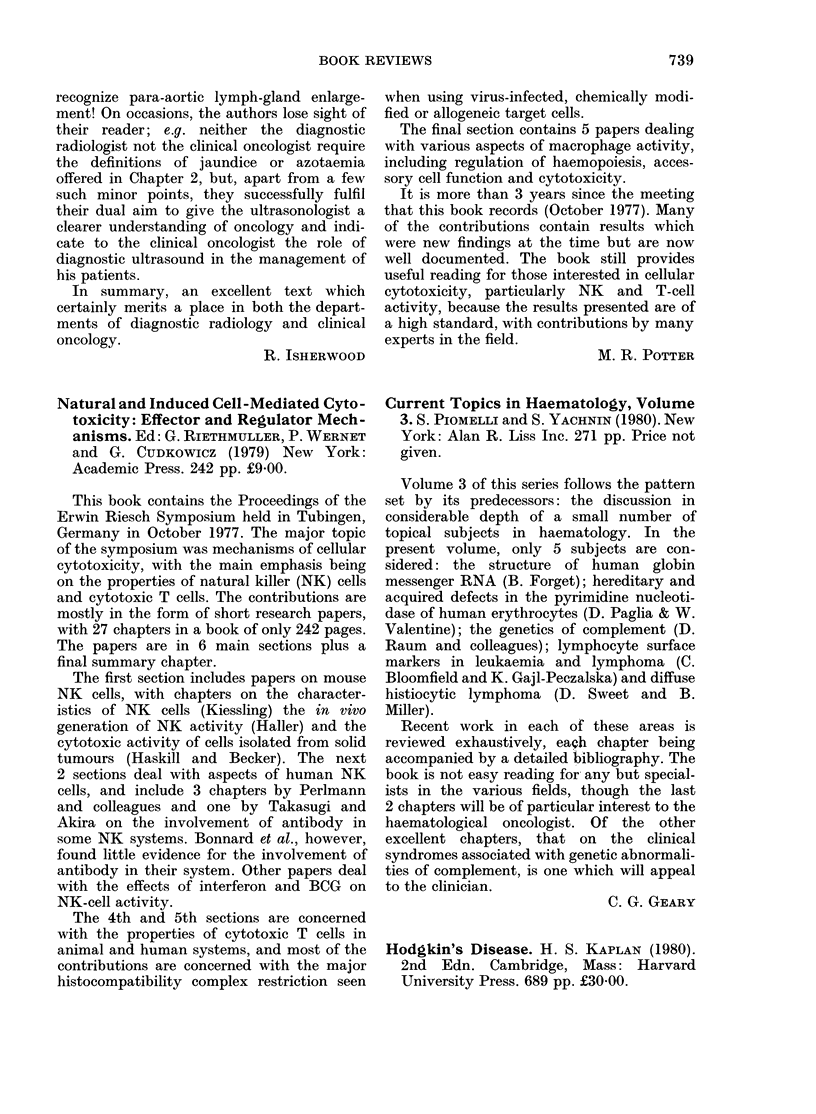# Natural and Induced Cell-Mediated Cytotoxicity: Effector and Regulator Mechanisms

**Published:** 1981-05

**Authors:** M. R. Potter


**Natural and Induced Cell-Mediated Cytotoxicity: Effector and Regulator Mechanisms.** Ed: G. RIETHMULLER, P. WERNET and G. CUDKOWICZ (1979) New York: Academic Press. 242 pp. £9·00.

This book contains the Proceedings of the Erwin Riesch Symposium held in Tubingen, Germany in October 1977. The major topic of the symposium was mechanisms of cellular cytotoxicity, with the main emphasis being on the properties of natural killer (NK) cells and cytotoxic T cells. The contributions are mostly in the form of short research papers, with 27 chapters in a book of only 242 pages. The papers are in 6 main sections plus a final summary chapter.

The first section includes papers on mouse NK cells, with chapters on the characteristics of NK cells (Kiessling) the *in vivo* generation of NK activity (Haller) and the cytotoxic activity of cells isolated from solid tumours (Haskill and Becker). The next 2 sections deal with aspects of human NK cells, and include 3 chapters by Perlmann and colleagues and one by Takasugi and Akira on the involvement of antibody in some NK systems. Bonnard *et al.*, however, found little evidence for the involvement of antibody in their system. Other papers deal with the effects of interferon and BCG on NK-cell activity.

The 4th and 5th sections are concerned with the properties of cytotoxic T cells in animal and human systems, and most of the contributions are concerned with the major histocompatibility complex restriction seen when using virus-infected, chemically modified or allogeneic target cells.

The final section contains 5 papers dealing with various aspects of macrophage activity, including regulation of haemopoiesis, accessory cell function and cytotoxicity.

It is more than 3 years since the meeting that this book records (October 1977). Many of the contributions contain results which were new findings at the time but are now well documented. The book still provides useful reading for those interested in cellular cytotoxicity, particularly NK and T-cell activity, because the results presented are of a high standard, with contributions by many experts in the field.

M. R. POTTER